# Borax for Colds

**Published:** 1879-10

**Authors:** 


BORAX FOR COLDS.—A writer in *The Medical Record* cites a number of cases in which borax has proved a most effective remedy in certain forms of colds. He states that in sudden hoarseness or loss of voice in public speakers or singers, from colds, relief for an hour or so, as by magic, may be often obtained by slowly dissolving, and partially swallowing a lump of borax the size of a garden pea, or about three or four grains, held in the mouth for ten minutes before speaking or singing. This produces a profuse secretion of saliva, or "watering" of the mouth and throat—probably restoring the voice or tone to the dried vocal cords, just as "wetting" brings back the missing notes to a flute, when it is too dry.

